# 

**Published:** 1872-10

**Authors:** 


### INDEX OF SUBJECTS.

### ORIGINAL COMMUNICATIONS.

Functional Diseases of the Eye,........................................ 577
Eclampsia—Purperal Convulsions........................................ 590
Chronic Cystitis—Perineal Cystotomy........................................ 598

### EXTRACTS AND GLEANINGS.

Diet for Infants—Chloral for Toothache, 600; Expectorant Mixture—Iodine in Vomiting, 601; Treatment of True Croup—Ivy Poisoning, 602; The Mumps—Chorea, 603; Vomiting of Pregnancy—Treatment of Enurosis, 605; Electrolysis in Ovarian Tumors—Eucalyptus, 606; For Enlarged Glands—Calabar Bean in Cerebro Spinal Meningitis, 607; Removal of Corns—For Rheumatism, 608; For Mercurial Somatitis—Mouth Wash, 609; Pepsine in Diarrhœa, 610; Syrup of Coffee—Bone Felon Arrestad—Emetia—Chloral, 611; Calabar Bean in Epilepsy—Dried Beef Pulp—Strychnia in Vomiting, 612; A Case of Calculus Nephritis—Treatment of Diabetes—Coryza, 613; Koussin in Tapeworm—Bullock's Blood, 614: Injection of Ammonia—Cure for Intemperance, 616; Xylol—Cocoanut Milk, 617, Epileptiform Tic Douloureux, 618; Enlargement of the Spleen, etc., 620; Blisters in Pleumonia—Balsamic Cigarettes for Asthma—Chloral as a Topical Application in Eczema, 621; Strychnia and Phosphoric Acid in Depression, 622; Case of Obstinate Vomiting—Penetrating Wounds of the Knee Joint—Removal of Plaster of Paris Bandages, 623; Case of Diabetes Treated by Carbolic Acid—Treatment of Hypertrophied Tonsils, etc.—The Oxalate of Potash, etc., 624; Dysentery—Phosphorus Pills—Veratria in Pneumonia—For Offensive Breath, 625.

### EDITORIALS, REVIEWS, ETC.

Dr. Swan M. Burnett—Dr. Alban S. Payne—Dr. Battey's Case, etc.,............ 626
Delinquents—Editorial Department........................................ 627
Suggestions and Kind Words from Friends........................................ 628
Literary Notices........................................ 636

